# The double-edged functions of necroptosis

**DOI:** 10.1038/s41419-023-05691-6

**Published:** 2023-02-27

**Authors:** Keng Ye, Zhimin Chen, Yanfang Xu

**Affiliations:** 1 Department of Nephrology, Blood Purification Research Center, the First Affiliated Hospital, Fujian Medical University, Fuzhou, 350005 China; 2 Research Center for Metabolic Chronic Kidney Disease, the First Affiliated Hospital, Fujian Medical University, Fuzhou, 350005 China; 3 Central Laboratory, the First Affiliated Hospital, Fujian Medical University, Fuzhou, 350005 China

**Keywords:** Cell growth, Necroptosis

## Abstract

Necroptosis refers to a regulated form of cell death induced by a variety of stimuli. Although it has been implicated in the pathogenesis of many diseases, there is evidence to support that necroptosis is not purely a detrimental process. We propose that necroptosis is a “double-edged sword” in terms of physiology and pathology. On the one hand, necroptosis can trigger an uncontrolled inflammatory cascade response, resulting in severe tissue injury, disease chronicity, and even tumor progression. On the other hand, necroptosis functions as a host defense mechanism, exerting antipathogenic and antitumor effects through its powerful pro-inflammatory properties. Moreover, necroptosis plays an important role during both development and regeneration. Misestimation of the multifaceted features of necroptosis may influence the development of therapeutic approaches targeting necroptosis. In this review, we summarize current knowledge of the pathways involved in necroptosis as well as five important steps that determine its occurrence. The dual role of necroptosis in a variety of physiological and pathological conditions is also highlighted. Future studies and the development of therapeutic strategies targeting necroptosis should fully consider the complicated properties of this type of regulated cell death.

## Facts

- Necroptosis plays a role in a variety of physiological and pathological conditions.
- Necroptosis can be induced by the activation of death receptors, among which TNFR1 is the most extensively investigated. Several key nodes influence whether or not the cell adopts a necroptotic fate, including the post-translational modification status of RIPK1, the activation status of caspase-8, RIPK3 homodimerization through the RHIM domain, the MLKL phosphorylation status, NINJ-mediated plasma membrane rupture, and ESCRT-III-mediated plasma membrane repair.
- The necroptotic pathway, which is highly pro-inflammatory, is usually considered to be detrimental, and blocking this pathway represents a promising therapeutic strategy for inflammation-associated diseases.

## Open questions

- Which factors determine whether the effects of necroptosis on the host are beneficial or detrimental?
- What is the collaborative contribution of necroptosis and other cell death modes in development and disease?
- How can the complex nature of necroptosis be manipulated under different pathological conditions?

## Introduction

Programmed cell death (PCD) is a regulated endpoint encountered by cells during normal development as well as under stress conditions linked to tissue injury and disease pathogenesis. Although apoptosis was once considered to be the predominant form of PCD, other types are now known to exist. In 2000, Holler et al. [1] found that Fas triggered an alternative, caspase-8-independent cell death pathway in which the kinase RIP (RIPK1) serves as the effector molecule. Moreover, it was reported that tumor necrosis factor (TNF)-induced programmed necrosis was facilitated by TNFR-2 signaling and caspase inhibition, and may play a role in controlling viral infection [2]. In 2005, necroptosis was described as a novel, TNF-α-triggered, non-apoptotic form of cell death occurring in the absence of caspase-8 [3]. Necroptotic cell death shares all the main morphological features of necrosis, including organelle swelling, lack of nuclear fragmentation, plasma membrane rupture, and leakage of intracellular components [4]. Mechanistic studies have led to necroptosis being characterized as a distinct type of cell death based on its molecular signaling pathways. Necroptosis is mediated by the necrosome, which consists of mixed lineage kinase domain-like protein (MLKL), receptor-interacting protein kinase 1 (RIPK1), and RIPK3. Evidence to date suggests that necroptosis plays an important role in a variety of pathological conditions, such as acute kidney injury [5], sepsis [6], ischemia–reperfusion injury [7, 8], and neurodegenerative disease [9].

Conflicting research results regarding the role of necroptosis have led to the proposal that necroptosis might act as a double-edged sword in a myriad of pathophysiological conditions. For instance, necroptosis can both promote and inhibit tumor growth [10], depending on the type of cancer and whether necroptosis occurs in malignant cells or cells of the tumor microenvironment. Furthermore, some pathogens can block host cell necroptosis to allow their replication. Like apoptosis, necroptosis is a conserved defense mechanism that may serve to protect against invading pathogens by limiting the lifespan of infected cells. However, studies have tended to focus excessively on the destructive effects of necroptosis, and have largely ignored its more complicated features, which may influence the development of therapeutic approaches targeting this form of PCD. Here, we review current knowledge of necroptosis and summarize the five critical steps that determine its occurrence. We also highlight its dual role under physiological and pathological conditions, especially in response to neurodegenerative disease, infectious inflammation, non-infectious inflammation, and tumor progression.

## The mechanisms of necroptosis

Events that promote extracellular stress, such as ischemia–reperfusion injury, calcium overload, drug stimulation, osmotic stress, and heat stress [11–14], can induce necroptosis through several signaling pathways involving ligand–receptor binding, including TNF-α/TNFR [15], Fas ligand/FAS [16], interferon-gamma (IFN-γ)/IFNAR1 [17], double-stranded RNA/Toll-like receptor 3 (TLR3) [18], and double-stranded DNA/Z-DNA binding protein 1 (ZBP1) [19]. Specifically, osmotic stress promotes necroptosis by directly stimulating RIPK3 kinase activity through an increase in cytoplasmic pH mediated by the Na^+^/H^+^ exchanger SLC9A1 [13]. Heat stress also leads to necroptosis by activating ZBP1 through heat shock transcription factor 1 (HSF1). Activated ZBP1 binds to and phosphorylates RIPK3, which subsequently recruits and phosphorylates MLKL; phosphorylated MLKL oligomerizes and translocates to the plasma membrane, where it executes necroptosis by inducing membrane rupture. Necroptosis can result in circulatory failure, organ injury, and lethality [19]. Table 1 summarizes several classical pathways that can elicit necroptosis. Of these, the molecular mechanism underlying TNF-α/TNF receptor 1 (TNFR1)-mediated necroptosis is the most extensively investigated [20]. These studies have identified several key molecules and critical events in necroptosis.

**Table 1:** The major death receptors that induce necroptosis.

Death ligand	Death receptor	Primary mechanisms for inducing necroptosis	Ref
TNF	TNFR1	The binding of TNF-α to TNFR1 triggers multiple signaling pathways, including NF-κB, apoptosis, and necroptosis. The core of the necroptosis mechanism is the regulation of the formation of necrosome, a complex consisting of RIPK1, RIPK3, and MLKL	[15, 20]
FASL (CD95L)	FAS (CD95)	cIAP deficiency promotes the recruitment of RIPK1 and Fas when caspase-8 is blocked and enhances the formation of the cytosolic ripoptosome complex, which induces necroptosis	[16]
TRAIL	TRAIL1/2 (DR4/5)	The combination of TRAIL to DR4/5 subsequently binds to FADD through the intercellular DD domain, leading to the formation of DISC directly. Then induce necroptosis via non-canonical recruitment of RIPK1.	[147]
dsRNA (polyI:C)	TLR3	TLR3 and TLR4 activate RIPK3 and participate in ensuing necroptosis via TRIF or Myd88. The c-terminal RHIM motif is required for RIPK3 to interact with TRIF or Myd88. The RIPK3/TRIF signaling complex recruits and phosphorylates MLKL, inducing ROS accumulation and mediating TLR3- and TLR4-induced necroptosis	[63]
LPS	TLR4		
IFNα/β/γ	IFNAR1	In bone-marrow-derived macrophages type, I IFNα and IFNβ bind to their cognate receptor IFNα/β receptor subunit 1 (IFNAR1) to activate Janus kinase 1 and form the IFN-stimulated gene factor 3 (ISGF3) complex. The ISGF3 complex promotes the induction and activation of necrosomes and triggers necroptosis in a transcription-dependent pathway.	[17, 65]
Virus (viral dsDNA)	ZBP1 (DAI)	Cytoplastic nucleic acid sensor Z-DNA binding protein 1(ZBP1; also known as DAI) can identify viral dsDNA, promote the recruitment of RIPK3 to form necrosomes without RIPK1, and induce RIPK3-dependent necroptosis	[18, 66, 67]
TWEAK	Fn14	TWEAK-induced apoptosis through the activation of the Fn14 receptor, Caspase inhibitors prevent TWEAK-induced apoptosis but sensitize to necroptosis via the generation of reactive oxygen species.	[5]

*TNF* tumor necrosis factor, *TNFR1* tumor necrosis factor receptor 1, *FASL* CD95L, *FAS* CD95, *TRAIL* tumor necrosis factor-related apoptosis-inducing ligand, *TLR3* Toll-like receptor 3, *ZBP1* Z-DNA binding protein 1, *TWEAK* TNF-like weak inducer of apoptosis, *Fn14* fibroblast growth factor-inducible 14

**Fig. 1:**
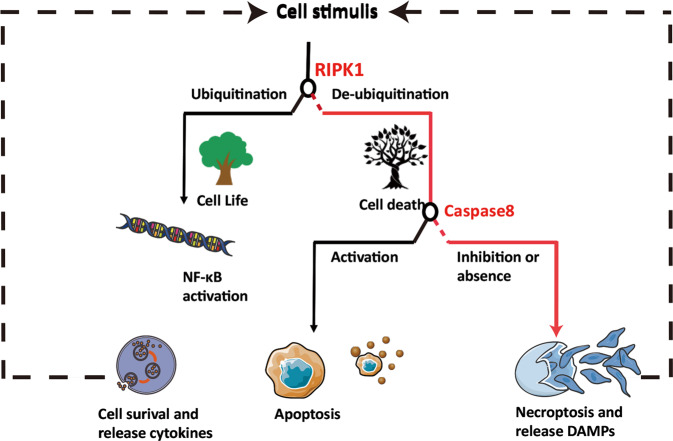
Several switch events exist in necroptosis. In TNFR1-induced necroptosis, cells undergo necroptosis only when RIPK1 is deubiquitinated, and caspase-8 is inhibited or absent. All death receptor-induced necroptosis requires the interaction of molecules containing the RHIM domain with the RHIM domain of RIPK3 to activate necroptosis.

Next, we review five critical steps in necroptosis using TNF-α/TNFR1-mediated necroptosis as the main example (Fig. 1).

### The post-translational modification status of RIPK1 is a master upstream regulator of necroptosis

**Fig. 2:**
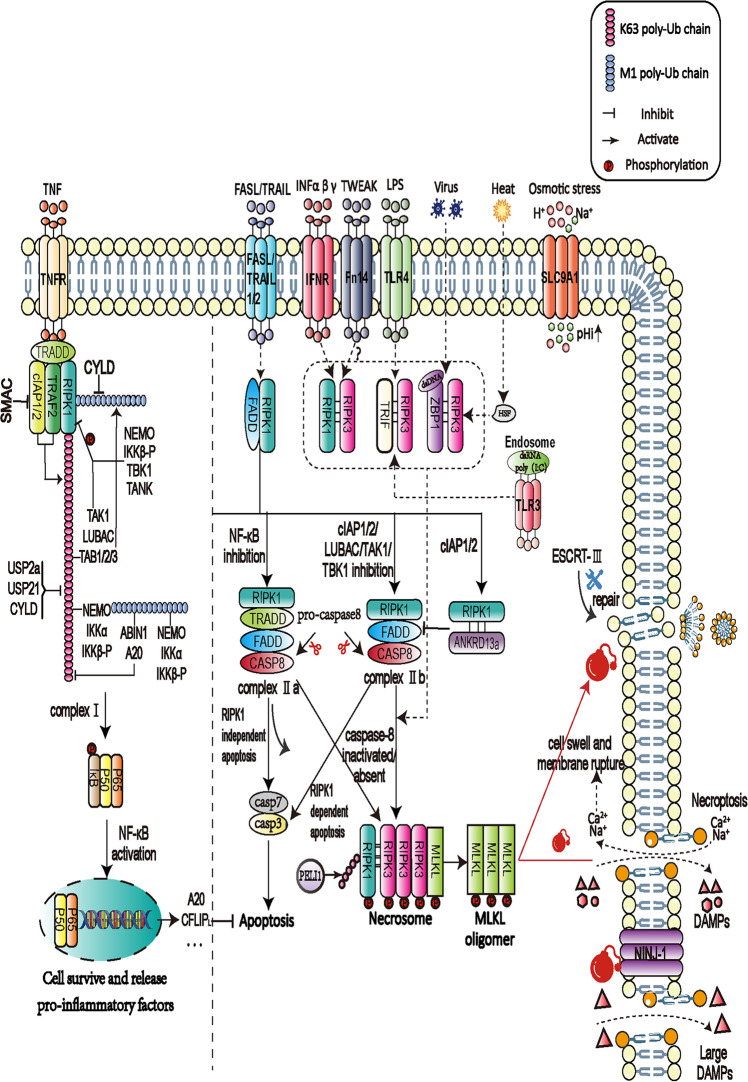
The molecular mechanisms of necroptosis. Tumor necrosis factor alpha (TNF-α) binds to TNF receptors, leading to the assembly of complex I, which is mainly composed of TRADD, RIPK1, CIAP1/2, and TRAF2/5. The ubiquitination status of RIPK1 determines the fate of complex I. In most cases, complex I recruits the TAK and IKK complexes, leading to the activation of the NF-κB pathway, which promotes inflammatory factor production and cell survival. When CYLD deubiquitinates RIPK1, A20, USP21, or USP20, both TRADD and RIPK1 detach from complex I and assemble into complex IIa with FADD and caspase-8, leading to apoptosis. RIPK1 can also form complex IIb with FADD and caspase-8. ANKRD13a restricts the interaction of FADD and caspase-8 with ubiquitinated-RIPK1 by binding to the latter via its Ub-interacting motif. When caspase-8 is inhibited or absent, RIPK1 binds to RIPK3 via the RHIM domain, leading to RIPK3 phosphorylation and necrosome formation. An increase in cytoplasmic pH mediated by the activity of the Na^+^/H^+^ exchanger SLC9A1 and osmotic stress promotes necroptosis by directly stimulating the kinase activity of RIPK3. Heat stress activates ZBP1 through the transcription factor HSF1, which binds to and phosphorylates RIPK3, leading to the induction of pyroptosis through the MLKL pathway. Subsequently, MLKL is phosphorylated and oligomerized, translocates to the cell membrane, and promotes membrane rupture. Multiple death receptors can mediate the occurrence of necroptosis (see Table 1). NINJ1 is only partially involved in the release of Lactate Dehydrogenase (LDH, a standard measure of PMR) during MLKL-dependent necroptosis. In addition, ESCRT-III can repair plasma membrane rupture downstream of MLKL.

TNF is a key component of the innate immune response. Upon binding to its receptor, TNFR1, TNF promotes the production of other cytokines via a membrane-bound complex (complex I) composed of TNFR1-associated death domain (TRADD), TNFR-associated factor 2 (TRAF2), RIPK1, cIAP1/2, and linear ubiquitin chain assembly complex (LUBAC) [21] (Fig. 2). Cells face three different outcomes after complex I formation: survival, apoptosis, or necroptosis. The post-translational modification status of RIPK1, which mainly includes ubiquitination and phosphorylation, has an essential role in directing the fate of a cell toward survival or death [22, 23].

The polyubiquitination of Lys63 in the intermediate domain of RIPK1 can be catalyzed by the complex I components cIAP1/2 and LUBAC [24, 25]. Polyubiquitinated RIPK1 can then serve as a scaffold for the IKK complex, composed of NEMO, IKKα, and IKKβ; and the TAK complex, composed of tat-associated kinase 1 (TAK1) and TAK1-binding protein (TAB)1/2 [26, 27]. The nuclear factor-kappa B (NF-κB) pathway can be activated by both the TAK and IKK complexes, promoting cell survival by stimulating the expression of numerous pro-inflammatory and pro-survival genes [28, 29]. When RIPK1 is deubiquitinated in certain situations, such as in the absence of LUBAC or cIAP1/2 via their binding to a second mitochondria-derived activator of caspase (SMAC) mimetics, TNF-α can induce cell death in the form of either apoptosis or necroptosis [30, 31].

The status of RIPK1 ubiquitination can also be affected by multiple deubiquitinating enzymes and the deubiquitination function of deubiquitinating enzymes can exert in different stages of necroptosis. Cylindromatosis (CYLD) and A20 are two crucial regulators of inflammatory signaling and cell death; both can remove the K63 polyubiquitin chain from NF-κB complex proteins, thereby promoting the formation of a second cytosolic complex (complex II) and initiate either apoptotic or necroptotic cell death via disparate mechanisms (described below) [32–34]. Moquin et al. [35] found that CLYD also promoted the activation of RIPK1 in the necrosome. However, A20 was also reported to inhibit the ubiquitination of RIPK3 and interrupt the formation of RIPK1–RIPK3 complexes, resulting in a deviation from necroptosis [36]. The ZnF7 ubiquitin-binding domain of A20 can prevent necroptosis in macrophages, where it has an anti-inflammatory effect [36]. Thus, the same molecule exerts its dual character in the process of necroptosis. SPATA2, an adapter protein, is recruited to complex I following TNFR1 activation and can regulate RIPK1 activation by modulating linear ubiquitination between LUBAC and CYLD [37]. PELI1, an E3 ubiquitin ligase, promotes the binding of activated RIPK1 to RIPK3 by mediating K63 ubiquitination on K115 of RIPK1 in kinase-dependent necroptosis [38]. Recently, Liu et al. [39] identified tankyrase-1 as a native component that, in response to a death stimulus, mediates complex II poly-ADP-ribosylation (PARylation). PARylation promotes the recruitment of the E3 ligase RNF146, which results in proteasomal degradation of complex II, thereby limiting cell death. Li et al. [40] identified a crucial ubiquitination site in the death domain (DD) of RIPK1—K627 in human RIPK1-DD and K612 in murine RIPK1-DD—that controls the overall ubiquitination pattern of RIPK1 and its DD-mediated interactions with other DD-containing proteins. The K627R/K612R mutation prevents RIPK1 activation and TNFR1 signaling-mediated apoptosis and necroptosis. The expression of the RIPK1 mutants D325V and D325H in mouse embryonic fibroblasts confers not only increased sensitivity to RIPK1 activation-mediated necroptosis but also induces the expression of pro-inflammatory cytokines such as IL-6 and TNF [41].

RIPK1 autophosphorylation at serine 166 plays a critical role in the activation of RIPK1-dependent apoptosis and necroptosis. Laurien et al. [42] demonstrated that S166 phosphorylation was necessary for the RIPK1-dependent pathogenesis of inflammatory pathologies in vivo in four relevant mouse models (colitis, hepatitis and cancer, skin inflammation, and TNF-induced systemic inflammatory response syndrome). Geng et al. [27] showed that sustained phosphorylation of the RIPK1 intermediate domain at multiple sites by TAK1 promotes RIPK1–RIPK3 interaction and, consequently, necroptosis. However, the phosphorylation of RIPK1 on Ser25 by IKKs plays a key role in directly inhibiting the kinase activity of RIPK1 and preventing TNF-mediated, RIPK1-dependent cell death [43]. TAK1, IKKα/β, and TANK-binding kinase 1 (TBK1)/IKK, which are recruited by the polyubiquitin chain generated after RIPK1 ubiquitination, can directly phosphorylate RIPK1, thereby inhibiting its kinase activity and promoting the NF-κB pathway, resulting in cell survival [44]. MAPK14 (p38α) and its substrate MAPKAPK2 (MK2) play essential roles in TNF-induced inflammatory cytokine production [45]. MK2 phosphorylates RIPK1, which prevents TNF-induced cell death [46]. RIPK1 phosphorylation is highly correlated with ubiquitination. Both of these post-translational modifications act as early checkpoints to protect cells from TNF-induced cell death.

### The inhibition of caspase-8 leads to necroptosis

When cell death becomes irreversible, an additional checkpoint determines whether the cell will commit to apoptosis or necroptosis. Caspase-8 plays an important role at this checkpoint [47]. Following the deubiquitination of RIPK1, complex I becomes destabilized, and a second cytosolic complex—complex IIa, consisting of TRADD, FADD, and caspase-8—forms, which leads the cell towards apoptosis. In this process, RIPK1 binds to FADD, which subsequently recruits pro-caspase-8, leading to the homodimerization of the latter, ultimately yielding mature caspase-8 [48, 49] (Fig. 2). In most cases, caspase-8 in complex IIa further activates caspase−3/−7, inducing apoptosis independently of the kinase activity of RIPK1 in complex IIa [50].

Under certain conditions, such as IAP or TAK1 suppression, another cytosolic complex—complex IIb, comprising RIPK1, RIPK3, FADD, and caspase-8—is assembled and orients the cell toward apoptosis. This type of apoptosis requires high levels of FLIPL as well as RIPK1 kinase activity [24, 51–53]. Ankyrin repeat domain-containing protein 13a (ANKRD13a) restricts the interaction of FADD and caspase-8 with ubiquitinated-RIPK1 by binding to RIPK1 via its Ub-interacting motif. Lys115 and Lys377 on RIPK1 are polyubiquitinated by cIAP1/2, which regulates how RIPK1 interacts with ANKRD13a as well as complex II formation [54]. When RIPK1 and RIPK3 overwhelm caspase-8, such as under conditions of high levels of RIPK3 and MLKL or when caspase-8 is inhibited by the pan-caspase inhibitor zVAD-fmk or viral cFLIPS mimics, complex IIb will transform into the necrosome, which comprises RIPK1, RIPK3, and MLKL. Yang et al. [55] recently found that PDK1 induces the activation of RSK1 via phosphorylation at the Ser221 site of the RSK1 NTKD domain. RSK1, a crucial kinase mediating caspase-8 phosphorylation, is then recruited to the necrosome, where it phosphorylates the Thr265 site of caspase-8, thus inactivating it and promoting necroptosis. During necrosome generation, RIPK3 links to RIPK1 via its RIP homotypic interaction motif (RHIM) domain, and the RIPK3–RIPK1 complex further recruits MLKL, which is the primary executor of necroptosis [15].

Although the roles of activated caspase-8 remain incompletely elucidated, there is evidence indicating that caspase-8 plays a major part in another type of PCD known as pyroptosis. TAK1 inhibition has been demonstrated to activate caspase-8 in the RIPK1/FADD/caspase-8 complex, leading to GSDMD cleavage and pyroptosis [56, 57]. During bacterial infection, caspase-8 has been shown to interact with ASC, the caspase-1-activating adapter protein [58]. Newton et al. [59] found that the C362A mutation in CASP8 in intestinal epithelial cells induces intestinal atrophy and perinatal lethality in mice not via the MLKL-mediated necroptosis pathway but by activating ASC and the protease caspase-1. CASP8 (C362A) regulates the life and death of mice by interacting directly or indirectly with caspase-1, caspase-11, and Ripk3. Hence, caspase-8 has been suggested to be a regulator of crosstalk between multiple cell death-related pathways [60].

### RHIM–RHIM interactions of RIPK3 are essential for the phosphorylation of MLKL

After necrosome formation, the kinase domain of RIPK1 promotes the activation of RIPK3 through *cis*-autophosphorylation, following which activated RIPK3 phosphorylates MLKL, thus mediating plasma membrane rupture. Intriguingly, studies have confirmed that RIPK3–RIPK3 homodimerization is sufficient to activate MLKL [61, 62]. The RHIM domains of RIPK1 and RIPK3 allow the interaction of the two proteins, which is critical for necroptosis. Signaling pathways other than the TNF-α/TNFR1 pathway also underscore the importance of the RHIM domain. Lipopolysaccharide, the major component of the outer membrane of Gram-negative bacteria, acts as a typical surface antigen and can bind to the death receptor TLR4 to recruit TRIF, an RHIM domain-containing cellular adapter protein. TRIF then mediates necroptosis by binding to the RHIM domain of RIPK3 in a RIPK1-independent manner [63–65]. Similarly, ZBP1, a cytoplasmic protein that can be activated after binding directly to viral dsDNA in the cytoplasm of infected cells, also contains the RHIM domain. Activated ZBP1 interacts with RIPK3 via their respective RHIM domains [18, 66].

A variety of stimuli can activate RIPK3 via RHIM–RHIM interactions; accordingly, a competitive relationship exists among RHIM domains inside the cell. When the RHIM domain of RIPK1 is mutated, ZBP1 strongly interacts with RIPK3 and induces RIPK3/MLKL-mediated necroptosis. The RIPK1 RHIM domain-dependent inhibition of ZBP1-mediated RIPK3 activation prevents perinatal death as well as skin inflammation in adult mice [67]. Moreover, a recent study revealed that murine cytomegalovirus protein M45, which contains an RHIM domain, can also block necroptosis by invading the RIPK1–RIPK3 complex by competing for the RHIM domain [68]. Zhang et al. [69] generated a point mutation in the V488P site of the C-segment of the RHIM structural domain of RIPK3 and found that cells expressing RIPK3^V448P^ were resistant to RIPK1-dependent apoptosis and necroptosis. Under heat stress, ZBP1 expression is promoted by HSF1, and activation of ZBP1, mainly through the RHIM domain, but not the Zα domain, subsequently mediates RIPK3-dependent PCD [14]. This explains why Zα-deficient ZBP1 can still mediate heat stress-induced RIPK3 activation and PCD despite being completely inactivated by viral infection. These observations highlight the potential of the RHIM domain as a therapeutic target for necroptosis-associated injury or disease.

### MLKL is the primary executor of necroptosis

RIPK3-mediated phosphorylation is thought to initiate MLKL oligomerization. Subsequently, oligomerized MLKL binds to phosphatidylinositol and cardiolipin, causing the entire necrosome to translocate from the cytoplasm to either the cell membrane or the organelle membrane, where it forms permeable pores, thereby disrupting membrane integrity and leading to necroptosis [70]. Samson et al. [71] identified MLKL trafficking and plasma membrane accumulation as crucial necroptosis checkpoints. The accumulation of phosphorylated MLKL at intercellular junctions accelerates necroptosis between neighboring cells, which may be relevant to inflammatory bowel disease and other necroptosis-mediated enteropathies. Plasma membrane rupture (PMR) is the final cataclysmic event in lytic cell death. Recently, the nerve injury-induced protein 1 (NINJ1)—a cell surface protein—is characterized as the ultimate mediator of PMR during various types of PCD. It has been reported that NINJ1 is required for the release of high mobility group box 1 (HMGB1, a known damage-associated molecular pattern [DAMP] and promoter of inflammation) and interleukin-1β (IL-1β) during pyroptosis-related PMR [72, 73]. However, NINJ1 is only partially involved in the release of lactate dehydrogenase (LDH, a standard measure of PMR) during MLKL-dependent necroptosis [15, 74].

### ESCRT-III plays crucial role in plasma membrane rupture and repair

Cells may withstand some degree of cell membrane rupturing, because cell membrane repair mechanisms can compensate for a certain amount of damage, which may eventually enable the cell to survive. These repair processes involve the endosomal sorting complex required for transport III (ESCRT-III) in the shearing and shedding of the cell membrane. ESCRT-III acts downstream of MLKL in the regulation of necroptosis [72]. Gong et al. [75] discovered the exact procedure by which ESCRT-III repairs cell membrane breakage in necroptosis in 2017. In addition to membrane repair, cells undergoing non-lethal membrane breakdown convert cell membrane stress signals into immune signals to alert the surrounding microenvironment. The non-lethal loss of cell membrane integrity can be considered a physical DAMP and PKCs phosphorylated at the S660 position function as pattern recognition receptors that mediate the downstream innate immune response. ESCRT-III system protein mutants (VPS4A^E228Q^ and CHMP3^1–179^) that inactivate ESCRT-III or genetically knock down the expression of CHMP3, VPS4A, and VPS4B can significantly affect membrane repair function and increase the release of IL-1β [76]. However, the ESCRT-mediated repair of perforin pores in the membrane may restrict the accessibility of the target cytosol to cytotoxic T lymphocyte-secreted granzyme, thus promoting the survival of cancer cells under cytotoxic attack [77].

### Necroptosis under physiological conditions

When cells are exposed to extreme physical or chemical environments, such as lasers, detergents, bacteria-derived membrane perforators, or immune cells, cell membrane integrity is lost, and the cell undergoes lysis from within, through mechanisms such as programmed cell necrosis, necroptosis, and pyroptosis [78].

Apoptosis and autophagy have been shown to play important roles in a variety of vital physiological processes, such as the shaping of developing organs and the healing of injured cells. The current consensus regarding the effects of necroptosis may overlook its role in mammalian growth and development [79, 80]. The lack of the necroptotic machinery components RIPK3 and MLKL is not lethal in mice, and affected mice can mature into fertile adults without any obvious phenotype; accordingly, it may be tempting to overlook the role of necroptosis in mediating proper embryonic development [81]. RIPK1/RIPK3/MLKL-dependent necroptosis promotes the aging of the male reproductive system in the mouse. RIPK3 suppression is particularly important for inhibiting necroptosis in the mouse testis. The necroptosis marker phospho-MLKL can be detected in the testes of elderly men, but not in those of young men [82, 83]. On the other hand, Webster et al. did not detect any differences in testicular pathology [84]. Stockley et al. [85] found that organisms that possess an MLKL ortholog have statistically significantly smaller litter sizes and longer interbirth intervals, indicating that necroptosis may be a quality control mechanism to ensure the vitality of precious offspring among vertebrates. The ubiquitination status of RIPK1 is a key determinant of its function and impaired ubiquitination of RIPK1 exerts a critical negative effect on embryogenesis. RIPK1 knockout mice and mice with mutations in the RIPK1 RHIM domain exhibit early postnatal lethality due to increased systemic inflammation and cell death. Tang et al. [81] found that early embryonic mortality resulted due to widespread cell death caused by the disruption of K63-linked ubiquitination on residue Lys376 of RIPK1. During neonatal development, the phosphorylation of MLKL-Ser82 prevents RIPK3-mediated MLKL activation, thereby preventing autoinflammation and spontaneous necroptosis [86].

Furthermore, animals lacking RIPK3 are more vulnerable to certain types of viral infection. RIPK3 and RHIM domain signaling can protect host cells against certain bacteria. Enteropathogenic *Escherichia coli* and similar rodent citrobacteria release specific proteases that cleave the RHIM structural domain, thereby infecting and destroying host intestinal cells. Nevertheless, RIPK3-dependent macrophage necroptosis caused by *Salmonella enterica* serotype Typhimurium or *Mycobacterium tuberculosis* promotes the spread of these pathogens. Similarly, an acute or chronic injury may also lead to RIPK3-dependent necroptosis and tissue damage [87]. Murphy et al. [88] identified the lymphocyte-specific protein tyrosine kinase (Lck) inhibitor, AMG-47a, as an inhibitor of necroptosis that interacts with both RIPK1 and RIPK3, with its ability to protect against cell death being shown to be dependent on the strength of the necroptotic stimulus. RIPK3-mediated protection may come at the cost of damaging host tissues.

Intriguingly, several recent studies have shed new light on our knowledge of necroptosis by identifying its role in tissue regeneration. Zhou et al. [89] demonstrated that necroptosis facilitates muscle regeneration by playing a key role in promoting muscle stem cell proliferation. In addition, Lloyd et al. [90] showed that necroptosis also exerts regenerative effects through the promotion of remyelination, while Ying et al. [91] found that MLKL-mediated breakdown of membrane myelin promotes nerve regeneration. These studies indicated that the activation of necroptosis might lead to surprisingly beneficial effects.

### Necroptosis in neurodegenerative diseases

Several studies involving transgenic mice have reported that RIPK1 and RIPK3 play a key role in mediating progressive axonal degeneration. For instance, optineurin was reported to suppress RIPK1-dependent necroptosis by regulating RIPK1 turnover. The loss of optineurin led to progressive necroptosis-mediated demyelination and axonal degeneration in the CNS [92]. Amyotrophic lateral sclerosis and frontotemporal dementia are neurodegenerative diseases with a common genetic susceptibility [93], with the development of both conditions having been associated with *TBK1* dysregulation. Ito et al. [92] found that in several neurodegenerative diseases, RIPK1 activation promotes microglia activation via RIPK1-dependent apoptosis or necroptosis, leading to neuroinflammation. RIPK1 activity can be effectively inhibited by direct differential TAK1-mediated phosphorylation [27]. Xu et al. [94] reported that, like TAK1, TBK1 inhibits RIPK1 activation by directly phosphorylating the T190 site of RIPK1. Reduced TAK1 expression due to aging, acting in conjunction with haploinsufficient mutations in TBK1, can activate RIPK1 and trigger aging-related neurodegenerative diseases. RIPK1 activation in microglia and astrocytes initiates a detrimental neuroinflammatory program that contributes to a neurodegenerative environment in progressive multiple sclerosis [95]. Mifflin et al. [96] identified a subclass of microglia in mouse models of ALS, which they termed RIPK1-regulated inflammatory microglia, that show significant upregulation of classical pro-inflammatory pathways. Onate et al. [97] demonstrated that necroptosis was activated in postmortem brain tissue from patients with PD and in a toxin-based mouse model of the disease.

The necroptosis of some cell types is associated with demyelination and neurodegeneration; however, Lloyd et al. [90] showed that necroptosis also plays a regenerative role by inhibiting pro-inflammatory microglial activation, thus aiding remyelination. The authors suggested that reducing pro-inflammatory microglia mortality may constitute a unique method for reducing chronic CNS inflammation and stimulating a regenerative response that allows the restoration of myelin integrity. Moreover, Ying et al. [91] reported that MLKL-mediated breakdown of membrane myelin promoted nerve regeneration.

### Necroptosis in infectious inflammation

Necroptosis has emerged as both an instrument of innate immunity and an enhancer of inflammation in response to invasion by pathogens such as bacteria and viruses.

Gaba et al. [98] found that the NS1 protein of the human influenza A virus participates in necroptosis by interacting with MLKL. This interaction results in increased MLKL oligomerization and membrane translocation, the consequent activation of the NLRP3 inflammasome, and the subsequent release of IL-1β, which enhances antiviral defenses and results in virus clearance. SARS-CoV-2 infection can activate multiple cell death pathways, including necroptosis, apoptosis, and pyroptosis, in lung epithelial cells, which leads to an irremediable inflammatory cytokine storm and multi-organ failure [99, 100]. Unlike IAV and SARS-CoV-2, murine cytomegalovirus has evolved the ability to suppress necroptosis to extend its opportunity for replication within host cells [101, 102]. Liu et al. [103] identified an inhibitor of necroptosis in cowpox virus and other orthopoxviruses that exerted its effect by binding to RIPK3 and inducing its ubiquitination and proteasome-mediated degradation, thereby inhibiting necroptosis. ZBP1 (encoded by an interferon-stimulated gene), which harbors two RHIM domains, can also trigger necroptosis by recruiting RIPK3 and promoting RIPK3-mediated MLKL phosphorylation and consequent activation, which results in cell death. Numerous disease-causing viruses, including cytomegalovirus, herpes simplex virus, vaccinia virus, West Nile virus, Zika virus, and influenza A virus, have been linked to ZBP1-mediated cell death. The UL39-encoded protein ICP6 of HSV functions as a suppressor of RHIM-dependent RIPK3 activities in the natural human host. In contrast, ICP6 RHIM-mediated recruitment of RIPK3 in the non-natural mouse host directly activates necroptosis [104]. In addition, Dou et al. [105] found that necroptosis is enhanced by interferon-mediated suppression of miR-324-5p, thus contributing to antiviral defenses.

Under conditions of infection with a low bacterial load of *Staphylococcus aureus*, resulting in low-grade inflammation, apoptosis presents as a preferential mechanism for the clearance of the bacterium. However, the bacteria that survive the apoptotic stimuli can inhibit apoptotic signaling, resulting in high-level *S. aureus* infection and, consequently, a more deleterious “second wave” of cell death. Various toxins released by this bacterium can induce necroptosis and pyroptosis. Together with pyroptosis, necroptosis serves as the host reaction required to reduce inflammation and kill bacteria [106, 107]. Furthermore, robust glycolysis and the generation of reactive oxygen species in the mitochondria of host cells after prolonged infection with *S. aureus* small colony variants are sufficient to generate necroptosis. However, instead of eliminating the germs, active necroptosis increases the virulence of small colony variants [108]. Other bacterial infections, such as those caused by *Mycobacterium tuberculosis*, have also been linked to necroptosis activation [109]. Moreover, bacterial activation of the innate immune receptor TLR4 in the intestinal epithelium leads to gut barrier injury and an inflammatory microenvironment, and is required for the development of necrotizing enterocolitis [110]; however, the exact role of necroptosis in this process remains to be determined.

### Necroptosis in non-infectious inflammation

Necroptosis can trigger an inflammatory response even in the absence of infection. Lalaoui et al. [111] demonstrated the importance of caspase-mediated RIPK1 cleavage during embryonic development and showed that this event not only inhibited necroptosis but also maintained inflammatory homeostasis throughout life. RIPK1 acts as a transcriptional co-regulator in the nucleus and mediates the phosphorylation of SMARCC2, a key component of the BAF complex; RIPK1 also coordinates chromatin accessibility and is involved in the regulation of the transcription of genes that mediate the inflammatory response [112]. Yang et al. [113] showed how the upregulation of RIPK1 contributes to the pathogenesis of osteoarthritis by triggering chondrocyte necroptosis and ECM degradation through BMP7, a recently identified downstream target of RIPK1, in addition to MLKL. RIPK1 and RIPK3 have diverse functions in various liver disorders. Hepatocellular carcinoma and chronic inflammation are influenced by the RIPK1-related inflammatory response [114]. RIPK3 deficiency was shown to attenuate hepatic stellate cell activation and liver fibrosis in schistosomiasis through the downregulation of the JNK-cJUN/Egr1 axis [115]. Furthermore, in a study of necroptosis in renal ischemia–reperfusion injury, it was shown that Nec-1 attenuates cisplatin-induced acute kidney injury [116]. Our group further delineated some of the mechanisms underlying cisplatin-induced nephrotoxicity [5]. Using gene knockout or a chemical inhibitor, we showed that inhibition of RIPK1, RIPK3, or MLKL could diminish cisplatin-induced proximal tubule damage in mice [5]. RIPK3-deficient mice displayed reduced cell death in the pancreas, colon, and ileum [117].

Importantly, necroptosis may not be the only cause of some disorders. We previously found that GSDMD-mediated pyroptosis occurred in cooperation with RIPK3/MLKL-mediated necroptosis, which amplified inflammatory signaling and enhanced tissue injury in sepsis [6]. The BH3-like domains of GSDMD and MLKL are targets for Bcl-2, a key regulator of pyroptosis and necroptosis [118]. The combined effect of necroptosis and other cell death pathways has also been explored in inflammatory bowel diseases [119].

Intriguingly, necroptosis also exerts anti-inflammatory effects in some cases. Kearney et al. [120] found that, in HeLa, L929, MEFs, BMDMs, and HT-29 cells, RIPK3/MLKL pathway-mediated necroptosis led to a significant reduction in the synthesis and release of cytokines and chemokines following stimulation with TNF and lipopolysaccharide, thereby playing an inhibitory role in the host inflammatory response. The induction of necroptosis in TLR3-/4-stimulated microglia was shown to protect neurons against neurotoxic inflammation [121]. Nevertheless, the mechanism underlying the dual effects of necroptosis in inflammation remains to be explored.

### Necroptosis in tumors

Mounting evidence has indicated that the role of necroptosis in tumors is complex. Necroptosis may exert either pro- or antitumor effects, depending on the type of cancer and whether necroptosis occurs in malignant cells or cells of the tumor microenvironment (Table 2). In mammals, apoptosis is the first line of defense against tumor cells; however, apoptotic cell death is suppressed in a variety of cancers [122, 123]. In such cases, necroptosis can act as a powerful second line of defense in the clearance of tumor cells. The expression of key molecules in the necroptotic pathway is downregulated in different types of cancers, indicating that cancer cells may be killed by the activation of necroptosis [124–127].

**Table 2:** The pro- and anti-tumoral effects of necroptosis in tumors.

	Necroptotic cell type	Tumor type
Anti-tumoral effects	TCs	Breast cancer [128]
		Cervical squamous cancer [29]
		Hepatocellular carcinoma [130]
		Colorectal cancer [144]
		Head and neck squamous cell carcinoma [127]
Pro-tumoral effects	TCs	PDA [147]
	ECs	Melanoma、Breast cancer
		Lung carcinoma [148]
	Intestinal epithelial cell	Colorectal cancer [144]
	Hepatocytes	ICC [114]

*TCs* tumoral Cells, *ECs* endothelial cells, *PDA* pancreatic ductal adenocarcinoma, *ICC* intrahepatic cholangiocarcinoma

Activated necroptosis signaling has been proposed to exert protective effects against tumor malignancy. Shen et al. [128] showed that the levels of RIPK1, RIPK3, and MLKL were significantly upregulated in breast cancer tissues. The activation of RIPK3 resulted in the inhibition of TRIM28 in cancer cells and the enhancement of the antitumor microenvironment [129]. Necroptosis-inducing genes such as *Ripk1, Ripk3*, and *Mlkl* are associated with intra-tumoral CD3^+^ and CD8^+^ T-cell density and, consequently, the prognosis of numerous tumors [130]. Necroptosis is considered a highly pro-inflammatory and immunogenic mode of cell death as DAMPs are released following cell lysis [131]. Besides the necroptosis-specific killing effect, the released DAMPs can attract and activate dendritic cells, leading to the establishment of an immunogenic tumor microenvironment [132]. Activated dendritic cells present tumor antigens to naive CD8^+^ T cells, which promotes their activation and differentiation into cytotoxic T cells with immunocidal activity [29]. Activated cells recruit inflammatory cells to propagate inflammatory reactions, leading to tissue injury, which potentially feeds back to the necroptosis pathway [133–136]. Owing to the positive feedback between cell death and inflammation in necroptosis, the induction of necroptosis in apoptosis-resistant cancer cells is considered one of the most promising anticancer therapeutic strategies.

Growing evidence indicates that DAMPs released by necroptotic cells within the tumor microenvironment can promote angiogenesis, inflammation, cell proliferation, and the metastasis of some cancer cells [137–140]. The reduced expression of RIPK3 and resistance to necroptosis resulting from epigenetic and genetic changes may benefit the survival of cancer cells. 2-Hydroxyglutarate (2-HG), a product of mutant isocitrate dehydrogenase 1 (IDH1), binds to DNA methyltransferase 1 (DNMT1) and induces the hypermethylation of the RIPK3 promoter, thereby rendering tumor cells more resistant to necroptosis [141]. The reversal of such resistance is potentially vital for the treatment of tumors with IDH1 mutations. Najafov et al. [142] were the first to identify oncogenes, including *BRAF* and *AXL*, that could drive the loss of RIPK3 expression in cancer cells. Fukasawa et al. [143] suggested that RIPK3 promoter hypermethylation underlay the loss of RIPK3 expression in human small-cell lung cancer. However, in colon cancer, the loss of RIPK1 and RIPK3 expression was found not to be due to epigenetic DNA modification. Instead, Moriwaki et al. [125] reported that the transcription of RIPK1 and RIPK3 in human colon cancer tissues was influenced by hypoxia, suggesting that hypoxia may be a key determinant of RIPK1 and RIPK3 expression in solid cancers. A recent study revealed that the METTL3-mediated increase in the m6A modification level of TRAF5 was increased in infiltrated M2-polarized tumor-associated macrophages, resulting in impaired necroptosis and, finally, the acquisition of acquired oxaliplatin tolerance in patients with colorectal cancer [144]. The RNA-editing enzyme ADAR1 is emerging as a key contributing factor to resistance to immune checkpoint blockade therapy, preventing immune checkpoint blockade responsiveness by repressing immunogenic double-stranded RNAs. The depletion or mutation of ADAR1 resulted in Z-RNA accumulation and the activation of ZBP1, which culminated in RIPK3-mediated necroptosis [145].

Necroptosis, however, also exhibits detrimental effects that may promote aberrant cell proliferation and metastasis, thus accelerating the development of several malignancies [137, 146]. Seifert et al. [147] reported that the deletion of RIPK3 or the inhibition of RIPK1 protected against oncogenic progression in mice. The blockade of necroptosis was associated with the development of a highly immunogenic myeloid and T-cell infiltrate, leading to the reprogramming of the antitumor microenvironment. As tumor cells undergoing necroptosis may release soluble factors favoring peri-tumoral immune suppression that two cytokines, CXCL1 and SAP130, were identified to be downregulated in RIPK3-deficient pancreatic ductal adenocarcinoma [147]. Another study revealed that endothelial cell necroptosis can help tumor cell extravasation. Both extravasation and metastasis require the expression of amyloid precursor protein in tumor cells and that of its receptor, death receptor 6 (DR6), on endothelial cells [148]. However, as RIPK3 also plays several other roles besides its function in necroptosis [149], it is still not clear to what extent necroptosis promotes the progression of tumors in vivo.

## Conclusion

**Fig. 3:**
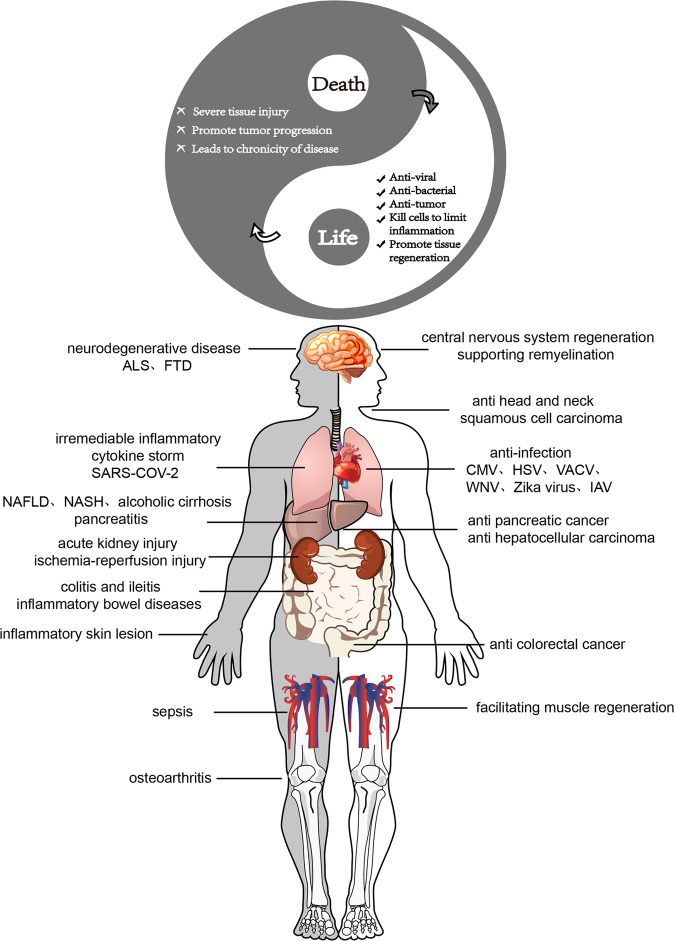
The “Tai Chi” model of necroptosis in regulating life and death. The adverse effects caused by necroptosis can be turned into positive effects; similarly, an excess of positive effects can lead to death in the host cells. Therefore, controlling the balance between life and death represents a considerable challenge.

Recent studies have indicated that necroptosis, a programmed cell death pathway with pro-inflammatory and immunogenic characteristics, plays a complex role in a variety of physiological and pathological conditions. It is now thought that necroptosis functions as a backup strategy in the event of apoptotic failure. Necroptosis exerts antiviral, antibacterial, and antitumor effects by removing infectious or proliferating cells and fostering the development of appropriate immunity. However, its potent cell-killing and pro-inflammatory effects can also lead to severe tissue damage, disease chronicity, and even tumor progression (Fig. 3). A growing number of studies have demonstrated the intricate relationship that exists between necroptosis and human disease; accordingly, the manipulation of critical components in the necroptosis signaling pathway may alleviate inflammation as well as mitigate pathological changes and tissue damage, all of which contribute to improved survival [79, 80]. A significant challenge to the therapeutic leveraging of necroptosis is that it is a relatively conservative defense mechanism that may be evaded due to the adaptive ability of pathogens and tumor cells. The key mechanisms of necroptosis under different pathological conditions are still not fully understood. Additionally, how cells measure cellular stress events to initiate cell death pathways and how the switching between different modes of cell death is controlled remains unknown. Despite the abundance of animal and cell-based studies exploring necroptosis, further investigation is warranted to assess the therapeutic efficacy of necroptosis manipulation. Future research into necroptosis should be undertaken using multi-disciplinary approaches, including the use of simulated organoids that can replicate the complexities of the human body as much as possible [150]. In conclusion, current data raise the hope that targeting necroptosis will provide promising therapeutic opportunities, and show that the complex nature of necroptosis needs to be comprehensively considered during the development of therapeutic approaches.

## Supplementary information

20230214 checklist

## Data Availability

There are no experimental datasets, given that this is a review article that is prepared based on a literature review.
